# Immunometabolic Network Interactions of the Kynurenine Pathway in Cutaneous Malignant Melanoma

**DOI:** 10.3389/fonc.2020.00051

**Published:** 2020-02-03

**Authors:** Soudabeh Rad Pour, Hiromasa Morikawa, Narsis A. Kiani, David Gomez-Cabrero, Alistair Hayes, Xiaozhong Zheng, Maria Pernemalm, Janne Lehtiö, Damian J. Mole, Johan Hansson, Hanna Eriksson, Jesper Tegnér

**Affiliations:** ^1^Unit of Computational Medicine, Department of Medicine, Centre for Molecular Medicine, Karolinska Institute, Stockholm, Sweden; ^2^Biological and Environmental Sciences and Engineering Division (BESE), Computer, Electrical, and Mathematical Sciences and Engineering Division (CEMSE), King Abdullah University of Science and Technology (KAUST), Thuwal, Saudi Arabia; ^3^Unit of Computational Medicine, Algorithmic Dynamics Lab, Department of Medicine Solna, Centre for Molecular Medicine, Karolinska Institute and SciLifeLab, Stockholm, Sweden; ^4^Navarrabiomed, Complejo Hospitalario de Navarra (CHN), Universidad Pública de Navarra (UPNA), IdiSNA, Pamplona, Sweden; ^5^MRC Centre for Inflammation Research, Queen's Medical Research Institute, University of Edinburgh, Edinburgh, United Kingdom; ^6^Department of Oncology-Pathology, Karolinska Institute, Stockholm, Sweden; ^7^Department of Oncology/Skin Cancer Center, Theme Cancer, Karolinska University Hospital, Stockholm, Sweden

**Keywords:** kynurenine pathway, cutaneous malignant melanoma, immune metabolic network interactions, 3-hydroxykynurenine, CD4+ T helper (Th) cells

## Abstract

Dysregulation of the kynurenine pathway has been regarded as a mechanism of tumor immune escape by the enzymatic activity of indoleamine 2, 3 dioxygenase and kynurenine production. However, the immune-modulatory properties of other kynurenine metabolites such as kynurenic acid, 3-hydroxykynurenine, and anthranilic acid are poorly understood. In this study, plasma from patients diagnosed with metastatic cutaneous malignant melanoma (CMM) was obtained before (PRE) and during treatment (TRM) with inhibitors of mitogen-activated protein kinase pathway (MAPKIs). Immuno-oncology related protein profile and kynurenine metabolites were analyzed by proximity extension assay (PEA) and LC/MS-MS, respectively. Correlation network analyses of the data derived from PEA and LC/MS-MS identified a set of proteins that modulate the differentiation of Th1 cells, which is linked to 3-hydroxykynurenine levels. Moreover, MAPKIs treatments are associated with alteration of 3-hydroxykynurenine and 3hydroxyanthranilic acid (3HAA) concentrations and led to higher “CXCL11,” and “KLRD1” expression that are involved in T and NK cells activation. These findings imply that the kynurenine pathway is pathologically relevant in patients with CMM.

## Introduction

Cutaneous malignant melanoma (CMM) is the most common form of melanomas and arises from a malignant transformation of melanocytes (1). The 5-year relative survival rate for patients with stage I CMM is more than 95%, while until recently, median survival was only 6–9 months in stage IV disease (1). Targeting the oncogenic mitogen-activated protein kinase (MAPK) pathway with small molecule inhibitors, i.e., BRAF and/or MEK inhibitors (MAPK-inhibitors; MAPKIs), has led to improved overall survival (OS) and progression-free survival (PFS) in patients with BRAF-mutant CMM (2).

It has been shown that patients with advanced CMM receiving immune checkpoint inhibitors (ICPIs), targeting the immunosuppressive programmed cell death 1 receptor (PD-1) have favorable response rate (33–44%). However, approximately two-thirds of patients do not have a satisfactory response to ICPIs (3–6). More recently, preliminary results in patients with metastatic melanoma indicate that combining indolamine 2, 3-dioxygenase 1 inhibitors (IDO1i) with either CTLA-4 inhibitor or PD-1 inhibitor (PD-1i) increases the effectiveness of these immunotherapies in CMM patients (7–9). Though, in (ECHO-301/Keynote-252), randomized controlled trial in patients with metastatic melanoma, combination treatment with an IDO1 inhibitor, epacadostat, and the PD-1i, pembrolizumab, did not show any improvement of survival compared to single therapy with PD-1i (10).

MAPK dysregulation led to differential antitumor immune response, which involves in Th1 signaling and cytotoxic effect (11, 12). The beneficial immunologic effects of MAPKIs have previously demonstrated. For instance, MAPKIs induces immunologic changes in tumor cells such as higher MHCs expression, and tumor-associated antigens (13, 14). MAPKIs also lowers the induction of immunosuppressive cytokines such as IL-10, VEGF, or IL-6 (15, 16). Interestingly, immunomodulatory effects of MAPKIs, in combination with immune checkpoint inhibitors, emerge as a promising strategy in cancer therapy (17).

On the other hand, targeting some metabolic substances synergizes with MAPK inhibition and delays the onset of drug resistance in melanoma (18). Therefore, targeting metabolic pathway might enhance the impact of MAPK inhibitors. Combining IDO1i, which is an effector of the Kynurenine pathway (KP), with immune checkpoint blockaders has been used in order to improve the effectiveness of these immunotherapies.

**Figure 1:**
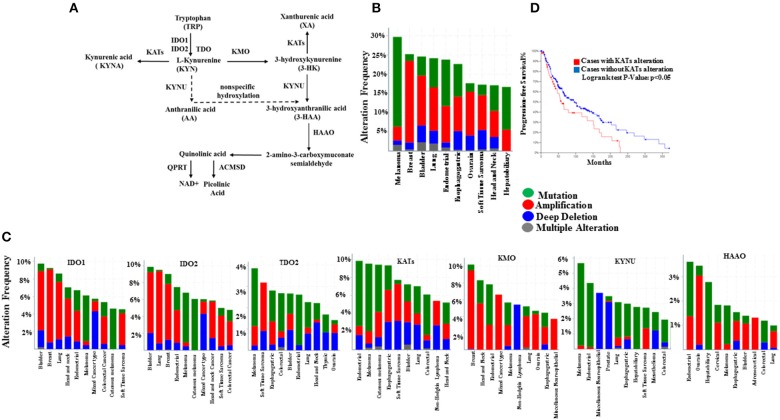
Genetic alterations of kynurenine pathway components in SKCM and other cancer types from TCGA dataset (cBioPortal). **(A)** The kynurenine pathway of tryptophan catabolism, **(B)** Frequency of genetic alterations in any of the kynurenine pathway enzymes, and **(C)** genetic alterations of genes encoding enzymes IDO1, IDO2, TDO2, KYNU, KMO, KATs (CCBL1, CCBL2, GOT2, and AADAT), HAAO, ACMSD, and QPRT for top 10 tumor types. DNA amplification (red), mutation (green), and deletion (blue) **(D)**, Kaplan–Meier survival plots for KATs alteration in 287 SKCM samples.

KP activation leads to tryptophan (TRP) metabolism and therefore generation of multiple immunomodulatory metabolites, known as Kynurenine pathway metabolites (KPM) including kynurenine (KYN), anthranilic acid (AA), kynurenic acid (KYNA), 3-hydroxykynurenine (3-HK), 3hydroxyanthranilic acid (3HAA), xanthurenic acid (XA) and culminates in the biosynthesis of nicotinamide adenine dinucleotide (NAD+) (Figure 1A) (19). The activity of the KP associated enzymes defined by the concentrations of direct derivatives of the metabolites, for instance the KYN: TRP ratio is used as an index of IDO activity (20–23).

KPMs have been measured using sensitive methods such as liquid chromatography-tandem mass spectrometry (LC-MS/MS). Except 3HAA, other KPMs are stable. 3HAA is known to be particularly unstable over time and sensitive to light (20, 24). It has been reported that accumulation of KYN induces T- B- and natural killer (NK)- cells apoptosis (25). The concentration of different metabolites in the KP is controlled by groups of enzymes such as IDO1, IDO2, and tryptophan 2, 3-dioxygenase (TDO2), three rate-limiting enzymes that catalyze the cleavage of TRP into KYN (26–28).

KYN itself is a substrate for different enzymes with immunomodulatory properties along KP. Enzymes such as kynurenine 3-monooxygenase (KMO) and kynurenine aminotransferases (CCBL1, AADAT, CCBL2, and GOT2; collectively called KATs) that convert KYN to 3-HK and KYNA, respectively (Figure 1A) (29–31).

It has been shown that KP activity is elevated in patients in numerous types of cancer (32–39). Moreover, the expression of IDO1 by tumor cells has been regarded as an essential immune escape mechanism that suppresses T cell activity (40, 41) through mTORC1 triggering autophagy (42, 43). Likewise, induction of IDO1 by inflammatory cytokines, including IFNγ and tumor necrosis factor α (TNF-α), has been linked to poor prognosis in CMM as well as various different cancer types (44, 45). It is also reported that plasma levels of KYN, 3-HK, and KYNA are associated with more aggressive forms of breast cancer and glioblastoma (38, 46). However, this has not been conclusively shown in CMM patients. Furthermore, whether KP metabolic dysregulation occurs in patients treated with MAPKIs was not known yet. Therefore, we undertook this study to evaluate immune-metabolic network interactions of the KP in CMM to explore the link between KPMs and regulation of the antitumor immune response. We measured the plasma levels of KPMs and immune-related proteins, using LC-MS/MS and proximity extension assay (PEA), respectively, in healthy individuals and, CMM patients before and during treatment with MAPKIs.

## Materials and Methods

### Cell Culture

Parental A375 BRAF V600E-mutated human melanoma cell line and daughter cell line with induced BRAFi resistance [vemurafenibR4 resistant subline (A375R)] were obtained from Professor Johan Hansson's research group.

Cell lines were cultured for 48 h for KPMs measurement at a density of 1.0 × 10^4^ cells/ml in RPMI 1640 medium (Gibco) supplemented with 10% heat-inactivated fetal bovine serum (Gibco) in the presence of IFNγ (50 ng/mL) and TNFα (10 ng/mL).

### Reagents

Recombinant IFNγ (cat. 285-IF-100) and TNF-α (cat. 210-TA7CF) were purchased from R&D system (McKinley Place NE).

### Cell Lines Authentication

We received early passages of parental A375 cells and the daughter BRAFi resistant subline and no changes in phenotype or morphology was observed. Furthermore, mycoplasma testing was routinely performed (LookOut Mycoplasma PCR Detection kit, Sigma Aldrich, MP0035), verifying that the cells were mycoplasma free (Supplementary Figure 10).

### Plasma Sampling and Sample Collection

Plasma samples were collected before (PRE) and during treatment between day 21–28 (TRM) from melanoma patients with disseminated (M1c and M1b) disease, undergoing first-line therapy with MAPKIs (Table 1, Supplementary Tables 1A, 3). Blood was obtained from five patients, collected in EDTA tubes and centrifuged at 1,500 × g for 10 min within 1 h after collection. Separated plasma was centrifuged at 2,400 × g for 15 min and was frozen at −70°C within 1 h of processing. Samples were then shipped overnight on dry ice for analysis. All samples were stored at −70°C until they were assayed. The sample collection was conducted by Good Clinical Practice/the Declaration of Helsinki with informed consent from all patients and was approved by the Stockholm Regional Ethics Committee, Karolinska Institute, Sweden.

**Table 1:** Clinical characterization of melanoma patients.

	**Age**	**Gender**	**Primary**	**BRAF status**	**PFS^a^ (days)**	**OS^b^ (days)**	**Disease stage**	**Treatment response**	**Type of treatment**
1	60	Male	CMM^c^	V600E	186	696	M1c^d^	PR^e^	BRAFi^f^
2	50	Female	CMM	V600E	170	637	M1c	PR	BRAFi
3	32	Male	UK^g^	V600E	148	248	M1c	PR	BRAFi
4	40	Male	CMM	V600E	101	203	M1c	PR	BRAFi,
5	43	Female	CMM	V600E	562	Alive	M1c	PD^h^	BRAFi and MEKi^i^

a *Progression-free survival (PFS)*.

b *Overall survival (OS)*.

c *Cutaneous malignant melanoma (CMM)*.

d *Cutaneous or, subcutaneous metastases (M1c)*.

e *Partial response (PR)*.

f *BRAF inhibitor (BRAFi; Dabrafenib/Vemurafenib)*.

g *Unknown (UK)*.

h *Progressive disease (PD)*.

i *MEK inhibitor (MEKi; Binimetinib)*.

### Healthy Volunteers

Plasma was collected from five healthy volunteers at the Karolinska University Hospital to be used as healthy controls, and all donors gave their informed consent before participating. Only volunteers over the age of 18 years were included in the control set (Supplementary Table 1B). Those with the presence of any of the following conditions were excluded: renal dysfunction, hepatic dysfunction (Child-Pugh score B or C), pregnancy or breastfeeding, immune-mediated inflammatory disease or medications, blood dyscrasia or anemia.

### Liquid Chromatography-Tandem Mass Spectrometry (LC-MS/MS) for Analysis of Tryptophan Metabolites

Plasma samples were diluted at a 1:1 ratio with 4% phosphoric acid (in HPLC grade H_2_O), and internal standard(s) was added as appropriate (e.g., 50 ng deuterated tryptophan; d5-TRP). To allow quantification, a calibration curve was made using an array of aqueous standards prepared by serial dilution (100, 50, 20, 10, 5, 2, 1, 0.5, 0.2, and 0.1 ng) of the following compounds: tryptophan, kynurenine, kynurenic acid, 3-hydroxykynurenine, anthranilic acid, 3hydroxyanthranilic acid, quinolinic acid and xanthurenic acid. These standards were suspended in 1% bovine serum albumin as a surrogate plasma matrix, and the internal standard(s) (e.g., 50 ng d5-TRP or d5-KA) were added before a 1:1 dilution in 4% phosphoric acid (in HPLC grade H_2_O). Each sample and the aqueous standard was transferred to a well in a 96-well analyte extraction plate (H_2_O Oasis HLB, 10 mg sorbent, 30 μm particle size) and vacuumed to dryness, washed and dried. Extracts were washed with H_2_O and eluted with 80% methanol (in HPLC grade H_2_O.) The elute dried by nitrogen gas flow at 60°C and 30 L/min. Dried extracts were re-suspended in 100 μl 30% methanol in HPLC grade H_2_O. Ten microliter volumes of each suspended extract were injected onto a column (Ace C18-PFP column; 100 × 2.1 mm internal diameter, 1.7 μm) using a Shimadzu Nexera MP UHPLC liquid chromatography system linked to a QTRAP 6500 mass spectrometer (Sciex). The flow rate was set at 0.4 ml/min at 40°C. The separation was carried out using mobile phase A-−0.1% formic acid (in HPLC grade H_2_O) and mobile phase B-−0.1% formic acid (in methanol). Data were acquired and processed using Analyst 3.0 software ABI (Sciex).

### Mass Spectrometry Analysis of Parental A375 BRAF V600E-Mutated Human Melanoma Cell Line and of Daughter Cell Lines With Induced BRAFi Resistance

The detail was described previously (47).

### Plasma Protein Detection

Plasma samples were analyzed using proximity extension assay (PEA) at the clinical biomarkers facility at SciLifeLab, Uppsala, Sweden. Ninety twohuman protein biomarkers were measured using Olink® ImmunoOnc I panel (Olink Proteomics, Uppsala, Sweden). Measurements were performed using 1 μL of each sample. In PEAs, a pair of oligonucleotide-conjugatedantibodies bind to their targeted protein in the samples. When the two probes are in close proximity, the oligonucleotides will hybridize in a pair-wise manner. The addition of a DNA polymerase leads to a proximity-dependent DNA polymerization event, generating a unique PCR target sequence. The target sequence is detected and quantified using a microfluidic real-time PCR instrument (Biomark HD, Fluidigm). The Olink protocol has been described in detail by Assarsson et al. (48). Data is then quality controlled and normalized using an internal extension control and an inter-plate control, to adjust for intro- and inter-run variation. The final assay readout is presented in Normalized Protein expression (NPX) values, which is an arbitrary unit on a log2-scale where a high value corresponds to higher protein expression. More details on the assay detection limits, intra- and inter-assay precision data are available on the manufacturer's website (www.olink.com) (48).

### TCGA Analyses

Mutations, copy-number alterations, and mRNA and protein expression profile of KP enzymes (IDO1/2, TDO2, KMO, KYNU, CCBL1/2, GOT2, AADAT, and ACMSD in TCGA (provisional) cohort data accessed at cBioPortal for Cancer Genomics in January 2018, http://www.cbioportal.org.

cBioPortal delivers genomic datasets for 24 cancer diagnoses (http://www.cbioportal.org/). The following tumor types were selected: adrenocortical carcinoma (*n* = 92), cholangiocarcinoma (*n* = 51), bladder urothelial cancer (*n* = 413), colorectal adenocarcinoma (*n* = 640), breast cancer (*n* = 1,105), glioma (*n* = 1,136), cervical cancer (*n* = 308), stomach adenocarcinoma(*n* = 664), uveal melanoma (*n* = 80), renal adenocarcinoma (*n* = 359), liver hepatocellular carcinoma (*n* = 442), lung adenocarcinoma (*n* = 1,097), lymphoid neoplasm(*n* = 48), myeloid neoplasm(*n* = 200), ovarian epithelial tumor (*n* = 606), pancreatic adenocarcinoma(*n* = 186), mesothelioma (*n* = 87), prostate adenocarcinoma (*n* = 499), cutaneous melanoma (*n* = 479), sarcoma (*n* = 265), testicular germ cells cancer(*n* = 156), thymic epithelial tumor (*n* = 124), thyroid carcinoma (*n* = 516), endometrial carcinoma (*n* = 605).

The cutaneous melanoma dataset for KP related analyses in melanoma patients was comprised of mRNA-seq data (data accessed at cBioPortal for Cancer Genomics in December 2017, http://www.cbioportal.org/) (Supplementary Table 2). Survival analyses were correlated with alterations (mutation, amplification, and deletion) in KP-related genes.

### Human Protein Atlas Analysis

Human protein atlas has been used for protein and RNA expression analyses of kynurenine pathway-related genes. The protein expression score is based on immunohistochemistry staining and is assigned manually by annotators (https://www.proteinatlas.org/about/assays+annotation#ihk), and the RNA-seq quantification data information is found here: https://www.proteinatlas.org/about/assays+annotation#rna). Image available from proteinatlas.org.

### Statistical Analysis

Principal component analysis (PCA) was computed using FactorMineR package for PCA https://cran.r-project.org/web/packages/FactoMineR/index.html. KPMs concentration of healthy, PRE and TRM groups was used for PCA. A *t*-test was used to compare KPMs in two groups of CMM patients. The adjustment methods include the Bonferroni correction (“Bonferroni”) in which the *p*-values are multiplied by the number of comparisons. All *P*-values were two-sided. All analyses were performed using R Statistical Software or Qlucore v3.2 (Qlucore, Lund, Sweden) bioinformatic software.

### Network Analysis

In the correlation network analysis, a node represents a KPMs or immune-related biomarker, and an edge is defined by statistically significant correlations between biomarkers in analyses of pre-treatment (PRE) samples. These values (cut-off of 0.5; is the highest level of correlation for which the network is not fragmented) were used to reconstruct networks in PRE and TRM CMM groups (before and during treatment) (49). We defined hubs as nodes with a high degree of centrality (high connectivity with other nodes) in PRE and TRM network. Nodes with a degree higher than the 80th percentile were considered as hubs. Network analysis was carried out with the network analysis tool of a cystoscope (3.4.0).

## Results

### Differential Expression of Kynurenine Pathway Enzymes Is Associated With Poor Survival in Patients With CMM

To gain further insight into mutations, copy-number alterations, and expression profiles of KP enzymes, we examined the TCGA database (data available at cBioportal.org). The TCGA melanoma cohort consists of 479 samples, and nearly 70% of them were CMM, stage II, and III from patients with no neoadjuvant therapy before tumor resection (Supplementary Table 2). Our analysis showed that within the TCGA cohort, metastatic melanoma and breast cancer are the top two cancer types with the highest burden of gene alterations in enzymes in the KP (Figure 1B, Supplementary Figure 1A). The highest proportion of KP gene mutations (>20%) were found in melanoma patients (Figure 1B). Within the kynurenine pathway, KAT members have the highest mutation frequency, at >8% (Figure 1C). Besides, we found that genetic alterations of KATs that mediate KYNA production from KYN were associated with a reduced survival rate in melanoma patients in the TCGA cohort (Figure 1D).

Further investigation using the Human Protein Atlas, CBio portal in healthy skin and CMM lesions (stage III and IV), show more abundant mRNA/protein expression of CCBL1 and CCBL2 among other KATs (Supplementary Figures 2A–C). Importantly, CCBL1 alteration is correlated to poor prognosis in CMM patients (*p* < 0.001), while KYNU is associated with a better outcome (*p* < 0.05), which can suggest that the KP enzymes possess a different intrinsic mechanism of action (Supplementary Figure 1B). Expression of KMO is not detected in healthy skin, as previously reported (50) or melanoma tumors; while the protein expression of KYNU is lower compared to CCBL1 and CCBL2 both in healthy skin and melanoma tumors (Supplementary Figures 2B,C).

Additional analyses by using a TCGA cohort was processed by the Broad Institute's pipeline (Firehose run “28 January 2016”: doi: 10.7908/C11G0KM9) consisting of 368 CMM samples showing that BRAF-mutant tumors retain distinct KP gene expression profiles compared to those with wild-type BRAF (Supplementary Figure 9, Supplementary Table 3). This result may suggest the existence of an association between BRAF mutation background and altered mRNA expression of KP members.

### Kynurenine Pathway Metabolite Profiles in PRE and TRM Metastatic CMM Patients Is Compared to Healthy Controls

**Figure 2:**
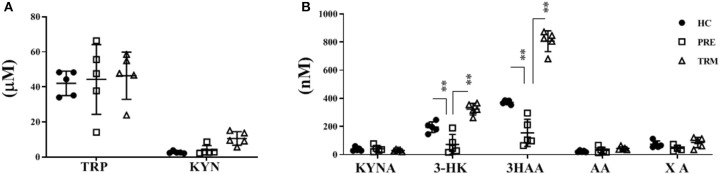
Kynurenine metabolites in plasma of CMM (PRE&TRM) compare to healthy controls. **(A)** The concentrations of KYN, TRP and **(B)** KYNA, 3-HK, AA, 3HAA, and XA were determined on plasma samples derived from healthy volunteers (HC, *n* = 5) and metastatic CMM patients, before (PRE) and during the first treatment (TRM) with MAPKIs (*n* = 5). TRP, tryptophan; KYN, L-kynurenine; KYNA, kynurenic acid; AA, anthranilic acid; 3-HK, 3-hydroxykynurenine; 3HAA, 3-hydroxyanthranylic acid; XA, xanthurenic acid; CMM, cutaneous; UM, Unknown Melanoma. **p < 0.005; Corrected *P*-Values (Bonferroni).

In order to explore how KP alteration in tumor biopsies reflected in the plasma and to further characterize the role of KP in CMM patients, we performed LC-MS/MS metabolite analysis on plasma samples derived from metastatic CMM patients, before (PRE) and during the first treatment (TRM) with MAPKIs (*n* = 5). Besides, plasma samples of five healthy volunteers were included in this comparison as controls. A total of seven metabolites, including TRP, KYN, KYNA, 3-HK, AA, 3HAA, and XA, were analyzed. The influence of confounding factors such as gender and, age on KP metabolite concentrations was compared between CMM patients and healthy controls using a principal component analysis (PCA) (Supplementary Figure 3). Our results were thus not affected by these confounders; therefore, they were not corrected in further analysis. Metabolic profile analyses revealed significantly lower 3-HK (*p* < 0.001) and 3HAA (*p* < 0.001) levels in PRE CMM-patients compared to healthy controls (Table 2, Figures 2A,B). Moreover, clinical intervention by MAPKIs was associated with increased concentrations of 3-HK and 3HAA in TRM plasma samples (Table 2, Figures 2A,B). These results suggest that 3-HK and 3HAA levels may serve as Predictive metabolites in CMM.

**Table 2:** Kynurenine metabolites in plasma of CMM (PRE&TRM) compare to healthy controls.

	**Healthy control (*n* = 5)**	**95% IC**	**PRE (mean *n* = 5)**	**95% IC**	**Corrected *P*-value (healthy vs. PRE)**	**TRM (mean *n* = 5)**	**95% IC**	**Corrected *P*-value (PRE vs. TRM)**
TRP (uM)	42.04	35.9–48.2	45.4	34.0–58.7	>0.9999	42.98	31.2–54.7	>0.9999
KYN (uM)	2.66	2.1–3.3	3.54	1.8–5.2	>0.9999	9.78	6.6–12.9	0.9374
KYNA (nM)	38.11	26.0–50.2	43.45	24.0–55.1	0.9880	27.57	19.8–35.4	0.9051
3-HK (nM)	195.30	162.6–228.0	73.23	21.9–111.2	<0.001^**^	324.9	295.6–354.2	<0.001^**^
3HAA (nM)	372.28	360.3–384.3	147.96	77.9–218.0	<0.001^**^	804.47	751.2–857.8	<0.001^**^
AA (nM)	23.10	17.9–28.3	28,51	13.1–40.5	0.3793	43.85	35.4–52.3	0.7467
XA (nM)	73.51	52.0–95.0	57.23	25.8–77.8	0.5776	88.51	62.1–115.0	0.3203

*TRP, tryptophan; KYN, L-kynurenine; KYNA, kynurenic acid; AA, anthranilic acid; 3-HK, 3-hydroxykynurenine; 3HAA, 3-hydroxyanthranylic acid; XA, xanthurenic acid (XA); CMM, coutenous; UM, Unknown Melanoma*.

** *p < 0.005; Corrected P-Values (Bonferroni)*.

Besides, a higher 3-HK/KYN ratio in healthy individuals indicates that more KYN metabolizes toward 3-HK in healthy controls compared to CMM patients. On the other hand, a higher XA/3-HK ratio was observed in melanoma patients, suggesting the higher activity of the KAYT II enzyme, which mediates the metabolism of 3-HK toward XA. Collectively, this result proposes that melanoma patients may have a higher accumulation of KYN and a lower concentration of 3-HK and 3HAA in the plasma. Therefore, KP possesses a different trajectory and path in healthy individuals compared with CMM patients (Supplementary Figure 6).

### MAPKIs Treatment and Association With Divergent Kynurenine Pathway Activities: Comparison of the Correlation Network in PRE and TRM Groups

**Figure 3:**
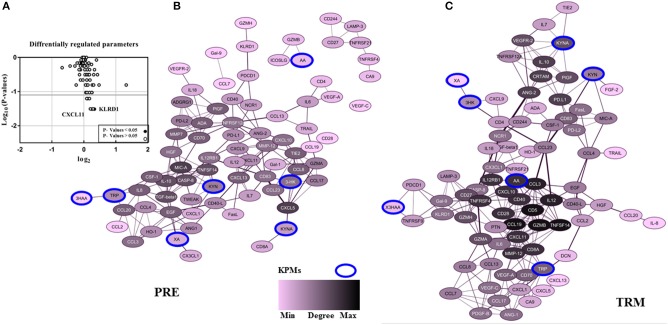
The pattern of CMM plasma protein and KPMs in PRE-and TRM groups. **(A)** differentially regulated proteins in the CMM patient PRE vs. TRM based on PEA measurement, **(B)** correlation networks between all PRE, and **(C)** TRM CMM patients network (cut-off: Spearman correlation ≥ 0.5). Nodes represents a molecular feature, and an edge specifies a statistically significant Spearman correlation between two markers (nodes).

**Figure 4:**
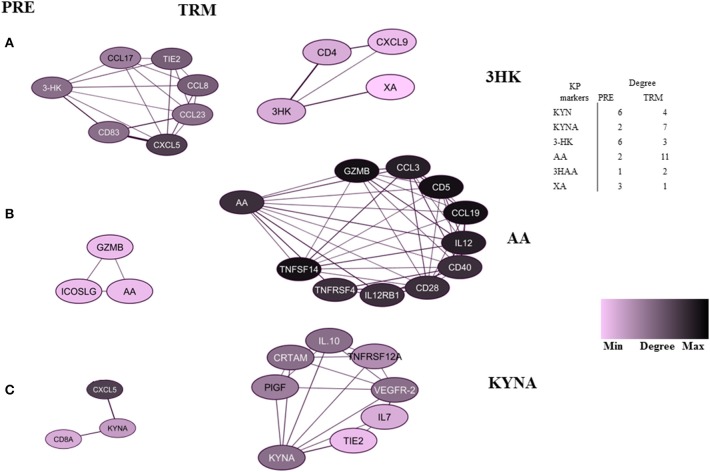
Highly-connected biomarkers which were at or above the 80th percentile of the degree distribution in CMM patients. **(A)** 3-HK, **(B)** AA, **(C)** KYNA. The table represents a summary network feature.

In the patient group, we evaluated whether there is an association between treatment and altered KPM concentrations/selected human plasma proteins (measured by LC-MS/MS and PEA using Olink® ImmunoOnc I panel, respectively) in PRE and TRM samples. The selected immuno-oncology PEA panel includes proteins involved in processes such as tumor promotion/suppression, tumor immunity, chemotaxis, vascular and tissue remodeling, apoptosis and cell killing and, metabolism. Reconstruction of correlation networks using Spearman correlation coefficient analyses were performed on plasma immune-related proteins and KPMs in PRE (Supplementary Tables 5, 7) and TRM groups (Supplementary Tables 6, 8). In this analysis, a node represents a KPM or immune-related biomarker, and an edge is defined by statistically significant correlations between biomarkers using correlation analyses. These values (cut-off: Spearman correlation ≥ 0.5; this is the highest level of correlation in which a network is not fragmented) were used to reconstruct networks in PRE and TRM CMM groups (49). Compared to the PRE network, the TRM network had more edges and higher median degree (Table 3A, Figures 3B,C, Supplementary Figure 7), which means nodes in TRM network tended to have a higher degree than those in the PRE-network (*p* = 0.007 based on a K-S test, Supplementary Figure 4A). This result indicates that distinct properties of the PRE and TRM network and changes in 3-HK levels in CMM plasma samples were associated with therapeutic intervention by MAPKIs. The median degree value was 4.0 in PRE-network and 6.0 in the TRM network. The total number of edges was significantly higher in the TRM network (*n* = 253) than in the PRE-network (*n* = 178) in a permutation test (*p* < 0.0001, Table 3, Supplementary Figure 4B). 3-HK has the highest number of connections in PRE and tended to lose interactions in the TRM group (Figure 4A).

**Table 3:** **(A)** Network characteristics **(B)** the overlapping between the hubs with differentially expressed parameters (PEA & LC-MS/MS) in PRE and TRM groups.

**Total (*N* = 12)**			**PRE (*N* = 5)**	**TRM (*N* = 5)**
**(A)**				
Correlation network based on original variables				
Number of nodes			81	82
Number of edges			178	253
Median degree			4	6
Average clustering coefficient			0.46	0.51
Hubs (degree centrality)			MIC-A(0.13)	GZMB(0.04)
			CASP8(0.06)	CD5(0.04)
			TNFSF14(0.06)	CCL19(0.04)
			MMP7(0.09)	TNFSF14(0.04)
			CD70(0.09)	IL-12(0.14)
			CXCL13(0.11)	CCL3(0.14)
			IL-10(0.001)	IL12RB1(0.12)
			TGFB1(0.06)	TNFRSF4(0.08)
			CCL8(0.05)	CXCL10(0.03)
			TIE2(0.05)	CD28(0.03)
			CXCL5(0.13)	CD40(0.03)
				AA(0.03)
				MMP12(0.04)
				CD8A(0.04)
				CXCL11(0.04)
**Names**	**PRE**	**TRM**	**Diffrentially expressed**	
**Total**	**11**	**15**	**4**	
**(B)**				
Elements	TIE2	IL-12	3-HK	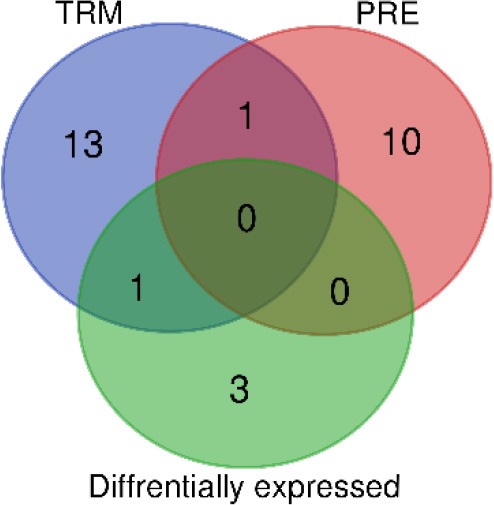
	TGFB1	CD40	KYN	
	CXCL13	MMP-12	KLRD1	
	CD70	TNFRSF4	CXCL11	
	CASP8	AA		
	CCL8	CCL19		
	MIC-A	CXCL10		
	MMP7	GZMB		
	IL-10	IL12RB1		
	CXCL5	CD5		
	TNFSF14	CD28		
		CCL3		
		CD8A		
		TNFSF14		
		CXCL11		

*Numbers are given of overlapping parameters compared to the total number. Venn diagram represents all shared and all unique markers in each group*.

On the other hand, AA and KYNA have the highest number of connections among the KPMs in the TRM network (Figures 4B,C). The value of the average clustering coefficient was 0.46 in the PRE network and 0.51 in the TRM network, indicating greater clustering tendency of the TRM network (Table 3). Together, 3-HK and KYNA interactions in the PRE-network identify the groups of proteins involved in the angiopoietin receptor Tie2 and the tyrosine phosphatase SHP2 signaling. Tie2 and SHP2 have inhibitory effect on Th1 differentiation (51–54); analysis was done by using functional annotation analysis of these sets of proteins with all gene sets in the Molecular Signatures Database (http://software.broadinstitute.org/gsea/msigdb/).

In particular IL-12, CD40, MMP-12, TNFRSF4, AA, CCL19, CXCL10, GZMB, IL12RB1, CD5, CD28, CCL3, CD8A, and CXCL11 were highly connected with other biomarkers in TRM network, but not in PRE-network (Table 3, Figures 3B,C, Supplementary Figures 5A,B). On the other hand, protein expression of KLRD1 (*p* < 0.05) and CXCL11 (*p* < 0.05) were higher than other proteins, measured by the PEA immuno-oncology panel, in TRM compared to PRE in CMM samples (Figure 3A, Supplementary Table 4). Despite insignificance of statistical power for the expression of CXCL9, CCL8, GZMA, and CCL3 (*p* = 0.06), the trend of higher expression in the mentioned proteins after treatment, worth attention, and evaluation in larger cohorts. Therefore, the interactions in the TRM network led to the identification of proteins that are involved in processes such as Th1/Th2 differentiation, IL12-mediated signaling events, and Toll-like receptor signaling; as shown by functional annotation analysis of this sets of proteins with all gene sets in the Molecular Signatures Database (http://software.broadinstitute.org/gsea/msigdb/).

Besides, we referred to mass spectrometry-based proteome analysis performed on parental A375 and the MAPKIs-resistant sublines (A375R) (Supplementary Figures 8B–D, Supplementary Table 9). These analyses show that KYNU expression was higher A375 cell line.

Furthermore, a set of highly correlated markers with KYNU is involved in activation of mTORC1 signaling pathway in this data sets (Supplementary Figure 8D). It is reported that TRP depletion leads to mTORC1 pathway suppression and consequently, cell-cycle arrest and T cell anergy (55–57). Thus, we speculated that the lower KYNU expression in A375R involves in acquisition of resistance to MAPKIs.

Moreover, we measured the KPMs concentration in A375 and A375R derived medium and cell lysate (Supplementary Table 10). Except 3HAA, all KPMs detected in the medium and in the cell lysate only TRP and KYN were detected. However, no differences were detected in the KPMs profile of these two cell lines, nor in the 48 h culture derived medium or in cell lysate (Supplementary Figure 8A).

We further investigated the KPM pathway alterations effect on the tumor/ immune cell. We explored whether Th1-cytokines, IFNγ and, TNF-α, can modulate the KYN downstream metabolites such as 3-HK, 3HAA, and AA in cancer cells. For this purpose, melanoma cell lines (A375 and A375R) were treated with IFNγ (50 ng/ml) and TNF- α (10 ng/ml). Although, 3HAA was not detected under these conditions and 3-HK levels was not affected by these cytokines, but IFNγ was able to induce AA in both cell lines (Supplementary Figure 8A).

Collectively, these results support that therapeutic intervention by MAPKIs leads to different kynurenine pathways metabolite trajectory which is of importance in altered Th1/Th2 differentiation markers in PRE and TRM CMM groups.

## Discussion

Much attention has been dedicated to determining how IDO1, which catalyzes the first and rate-limiting step of TRP degradation, modulates the immune response to tumors. Several clinical trials were designed to combine IDO1 inhibition with ICPIs (7–9). Despite expectations, a recent randomized phase 3 trial combining the IDO1 inhibitor, epacadostat, with pembrolizumab did not show any superior effect compared to pembrolizumab monotherapy in metastatic melanoma (10). This result indicates that the interplay between kynurenine biology and cancer is more complicated than was previously assumed. Besides decreased tryptophan/kynurenine ratio that has been associated with worse prognosis in several cancer types (58, 59), KP downstream metabolites could also play a role in tumoural immune escape. Therefore, inhibition of other components of the KP should be investigated in cancer therapy. Also, the complex interplay of the activity of the KP component may imply the existence of a feedback mechanism; e.g., KMO activity is one determinant of KYNA production by regulating the amount of KYN substrate available to KATs (60). However, the role of the KPMs, such as KYNA, 3-HK, and AA needs to be investigated further in cancers.

In this study, the cBioPortal with several hundreds of tumor biopsies obtained from different cancer types, including CMM, has been used. Intra-tumoural genetic alterations in KP enzymes have been reported in various cancer types. We also found that CMM and breast cancer patients have the highest KP related genes alteration. Moreover, CMM has the highest rate of KP mutations (>20%), specifically in KATs. Among KATs, CCBL2 is associated with poor survival in CMM patients. Furthermore, BRAF-mutant CMM retains distinct kynurenine pathway-related gene expression profiles compared to BRAF wild type, which suggests the existence of an association between mutation background and kynurenine pathway expression profiles.

Besides, we showed that KP metabolism is altered in PRE and TRM samples from CMM patients treated with MAPKIs, as reflected by changes in plasma levels of downstream metabolites. These new findings raise the question of whether altered kynurenine metabolism is a mean by which CMM cells escape immune attack. Interestingly, an altered ratio of KYNA: 3-HK was reported previously as an indicator of KP modulation in neurodegenerative disorders (61, 62).

By using correlation networks between KPMs and immune parameters, we identified 3-HK, AA, and KYNA as central nodes in network diagrams built around data from PRE and TRM patient samples. 3-HK and KYNA interactions in the PRE network identify groups of proteins such as CXCL13, IL-8, TIE2, ADGRG1, CCL20, and CXCL9 to be involved in Tie2 and SHP2 signaling with an inhibitory effect on Th1 differentiation (51–54) Interaction of Tie2, a tyrosine kinase receptor, with its ligand angiopoietin-1(Ang1), regulates angiogenesis which makes it an attractive target for cancer therapy (53). Furthermore, Ang1-Tie2 signaling suppresses T cell activation and promotes regulatory T cell expansion (52). Also, Tie2 regulation is involved in Th1 and CD8 immune responses (54). The tyrosine-protein phosphatase non-receptor type 11 (PTPN11), also known as SHP-2, is a critical intracellular regulator that mediates cell proliferation and differentiation (63). It is reported that SHP-2 suppresses Th1 immunity in the tumor microenvironment via the PD-1/SHP-2/STAT1/T-bet signaling axis (51).

On the other hand, correlation network analyses of samples from the same CMM patients showed that the TRM network was denser than the PRE network. Moreover, this analysis led to identification of proteins (CXCL11, IL-12, CD40, MMP-12, TNFRSF4, AA, CCL19, CXCL10, GZMB, IL12RB1, CD5, CD28, CCL3, and CD8A) to be highly connected with other biomarkers which are functionally enriched in processes such as Th1/Th2 regulatory switch, IL12-mediated signaling events and Toll-like receptor signaling.

These observations reveal the relative contribution of these proteins in T cell responses and show an association between higher 3-HK levels and CXCL11 and KLRD1 protein expression in CMM. CXCL11 itself is among the hubs in the TRM network (Table 3) and is a T cell activation marker. 3-HK is also connected to CD4+ and CXCL9 in TRM groups, the two factors that are involved in IL-23 signaling CXCL9 and CXCL10 also mediate the suppression of human CD4+ T cell activation by 3, 4-dimethoxycinnamonyl-anthranilic acid (tranilast) (64). While, KYNA connection to IL10, IL-7, and TNFRSF12A in TRM samples suggest an immune-regulatory role for KYNA (65, 66).

Additionally, higher expression of KYNU in A375 cell line compared with A375R was detected. Similarly, a set of highly correlated markers with KYNU is involved in the activation of mTORC1 signaling pathway, which plays a substantial role in the cell cycle and T cell proliferation (55–57).

Together, KP related enzymatic alteration in CMM tumor biopsies is associated with lower plasma level of 3-HK and 3HAA. MAPKIs therapy lead to induction of the 3-HK and 3HAA and is associated with Th1-like response.

Despite the small sample size, this is the first study that has assessed plasma KPMs-Immuno-oncology profiling of CMM patients. However, it is previously reported that lower level of KMO, 3-HK, and 3HAA is associated with less proliferative and exhausted CD4+ T-cells (67). In addition, Lee et al. presented a mild 3-HK increase is critical for cell death commitment and protects mitochondrial function, while more pronounced 3-HK increase induces the dysfunction of mitochondria and activate the apoptotic pathway (68). They additionally showed that 3-HK treatment reduced cell viability and co-treatment with ERK pathway inhibitor, PD98059, synergized in cell death induction. Similarly, active ERK decreased 3-HK induced-neural cell death and inhibition of ERK by specific ERK pathway inhibitor (PD98059) significantly impaired the 3-HK-induced cell death (68).

It is also reported that KYN potentiates the activity of the MAPK-ERK1/2 pathway (69), and inhibition of the MAPK pathway increases the serum levels of IFNγ and TNF-α (70). IFN-γ and TNF-α also induce KMO, KYNU expression as well as 3-HK production (71, 72). Therefore, we speculated that inhibition of MAPK pathway with MAPKIs impair the balance between MAPK pathways with KP activity and therefore activates compensatory feedback loops to elevate KP activation toward 3-HK and 3-HAA production.

Thus, the reduced KYNU expression in MAPKIs-resistant cell line (A375R) suggests that, this may be the rate-limiting step for the utilization of kynurenine. KYN directly activates aryl hydrocarbon receptors, resulting in the activation of immunosuppressive agents (19). Besides, strengthening KMO or KYNU activity leads to the utilization of KYN, and decreasing the excess amount of KYN in the plasma triggers immune evasion, a result that could restore immune surveillance (19). Hence, we speculate that treatments targeting KMO or KYNU may restore antitumor immunity.

However, our result contradicts those reports where the accumulation of KPMs such as 3-HK and 3HAA mediated cell apoptosis in T- B- and natural killer cells (NK) (25). Alternatively, Fallarino et al. reported that 3HAA would induce selective apoptosis in Th1 but not Th2 cells (73). They also suggest that the selective effects of KPMs on T cell apoptosis is not only are required for the maintenance of T cell homeostasis and self-tolerance, but also initiate specific disease conditions through an imbalanced T helper response, which does not necessarily disagree with our results.

In conclusion, this study provides a paradigm to investigate further how the many and seemingly different activities of KMPs could be expanded to accommodate a critical and possibly unifying role in immune regulation in CMM patient.

## Data Availability Statement

All datasets generated for this study are included in the article/Supplementary Material.

## Ethics Statement

The studies involving human participants were reviewed and approved by the sample collection was conducted by Good Clinical Practice/the Declaration of Helsinki with informed consent from all patients and was approved by the Stockholm Regional Ethics Committee, Karolinska Institute, Sweden. The patients/participants provided their written informed consent to participate in this study.

## Author Contributions

SR and JT: design, conception, and developed the methodology. SR, HM, NK, DG-C, and JT: analyzed and interpreted the data. AH, XZ, and DM: performed the HILIC-MS/MS. MP and JL: PEA data. SR, HM, NK, DG-C, AH, MP, JL, XZ, DM, HE, JH, and JT: writing and reviewing of the manuscript.

### Conflict of Interest

The authors declare that the research was conducted in the absence of any commercial or financial relationships that could be construed as a potential conflict of interest.
