# Supplementary material for: Immunometabolic Network Interactions of the Kynurenine Pathway in Cutaneous Malignant Melanoma
Source: Front Oncol. 2020 Feb 3;10:51. doi: 10.3389/fonc.2020.00051 (PMC7017805; doi:10.3389/fonc.2020.00051)
Supplement: Supplementary file 1 [file Table_1.docx]

| **Table S1**   1. Diagnostic parameters for melanoma | | | | | | | | | |
| --- | --- | --- | --- | --- | --- | --- | --- | --- | --- |
| **Pateitns (n=5)** | **LDH (mmol/L)**  **PRE** | **Leukocyte**  **(10(9)/L)** | | **Eosinophil**  **(10(9)/L)** | | **Lymphocyte**  **(10(9)/L)** | | **Neutrophil**  **(10(9)/L)** | |
|  |  | **PRE** | **TRM** | **PRE-** | **TRM** | **PRE-** | **TRM** | **PRE-** | **TRM** |
| 1 | 3.1 | 5.9 | 4.4 | 0.2 | 0.2 | 2.4 | 2.1 | 2.6 | 1.4 |
| 2 | 5.9 | 6.2 | 4.8 | 0.1 | NA | 1.5 | 1 | 3.9 | 2.8 |
| 3 | 9.8 | 17.3 | 5.9 | NA* | NA | NA | NA | NA | NA |
| 4 | 16 | 4.9 | 4.9 | <0.1 | NA | 1.3 | NA | 3.1 | NA |
| 5 | 3.2 | 14.9 | 8.3 | NA | NA | NA | NA | NA | NA |
| *NA Not availabe | | | | | | | | | |

1. The age of the healthy volunteers and CMM patients at the time of sample collection

|  | **CMM patients** | **healthy volunteers** |
| --- | --- | --- |
| 1 | 60 | 28 |
| 2 | 50 | 24 |
| 3 | 32 | 49 |
| 4 | 40 | 56 |
| 5 | 43 | 29 |
| **Mean** | 45 | 37.2 |

’
