# Supplementary material for: Immunometabolic Network Interactions of the Kynurenine Pathway in Cutaneous Malignant Melanoma
Source: Front Oncol. 2020 Feb 3;10:51. doi: 10.3389/fonc.2020.00051 (PMC7017805; doi:10.3389/fonc.2020.00051)
Supplement: Supplementary file 3 [file Presentation_1.pdf]

Supplementary Materials

Immunometabolic network interactions of the kynurenine pathway in metastatic cutaneous melanoma

A

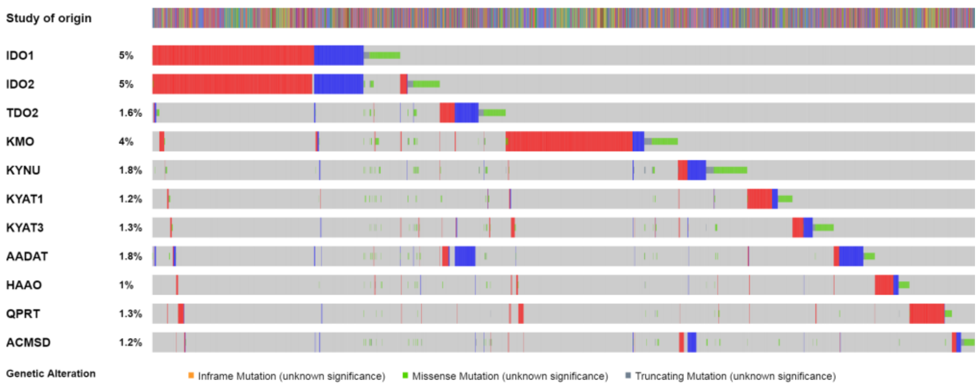

Supplemental Fig. S1. Genetic alterations of kynurenine pathway pathway components in SKCM from TCGA datasets

(A), OncoPrint showing genetic and expression alterations from 287 SKCM samples. genetic alterations of all kynurenine pathway enzymes IDO1, IDO2, TDO2, KMO, KYNU, CCBL1, CCBL2, GOT2, AADAT, HAAO, ACMSD and QPRT. DNA amplification (red), mutation (green), and deletion (blue) (B), Kaplan–Meier survival plots for IDO1, IDO2, TDO2, KMO, KYNU, CCBL1, CCBL2, GOT2, AADAT, HAAO, ACMSD and QPRTmutation in 287 SKCM samples.

B

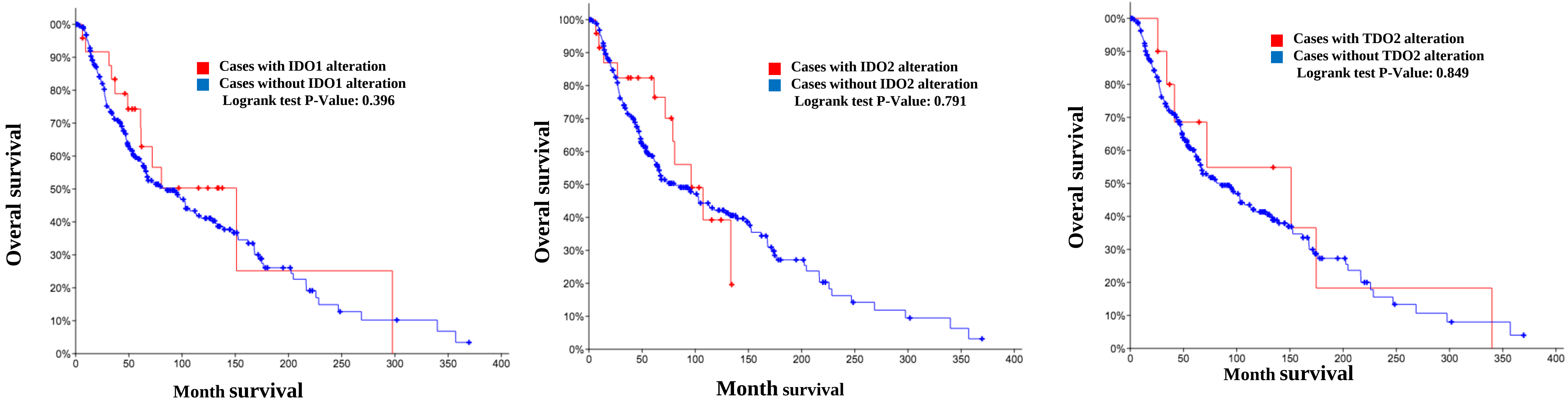

Supplemental Fig. S1

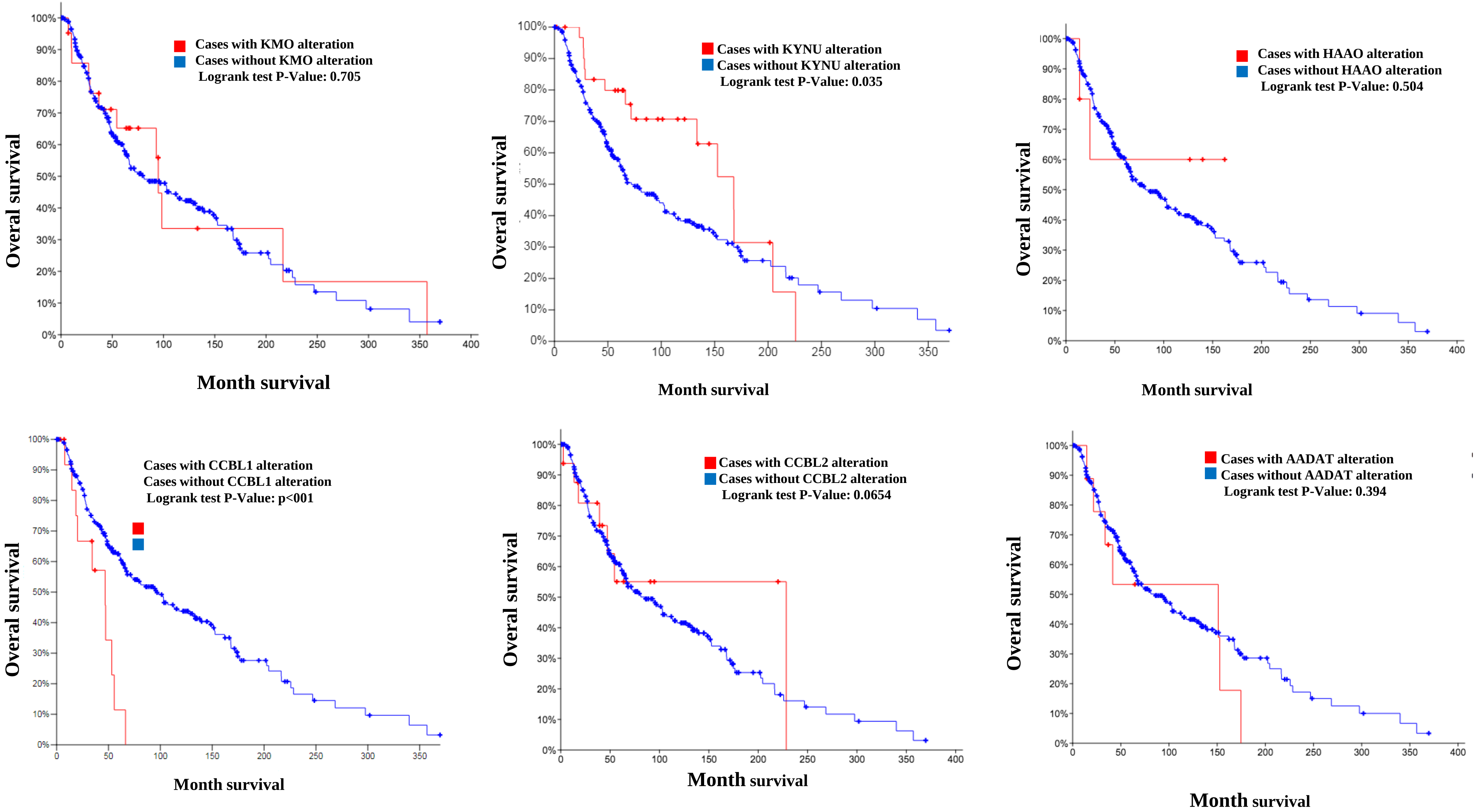

Supplemental Fig. S1

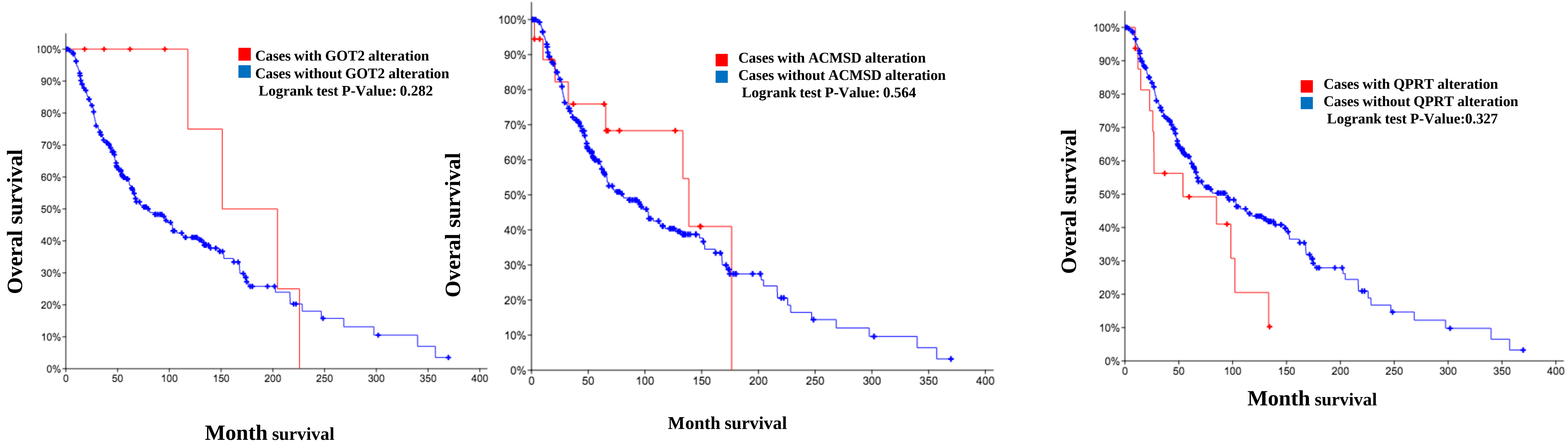

Cases with GOT2 alteration
Cases without GOT2 alteration
Logrank test P-Value: 0.282
Overal survival
Month survival
Cases with ACMSD alteration
Cases without ACMSD alteration
Logrank test P-Value: 0.564
Overal survival
Month survival
Cases with QPRT alteration
Cases without QPRT alteration
Logrank test P-Value:0.327
Overal survival
Month survival

A

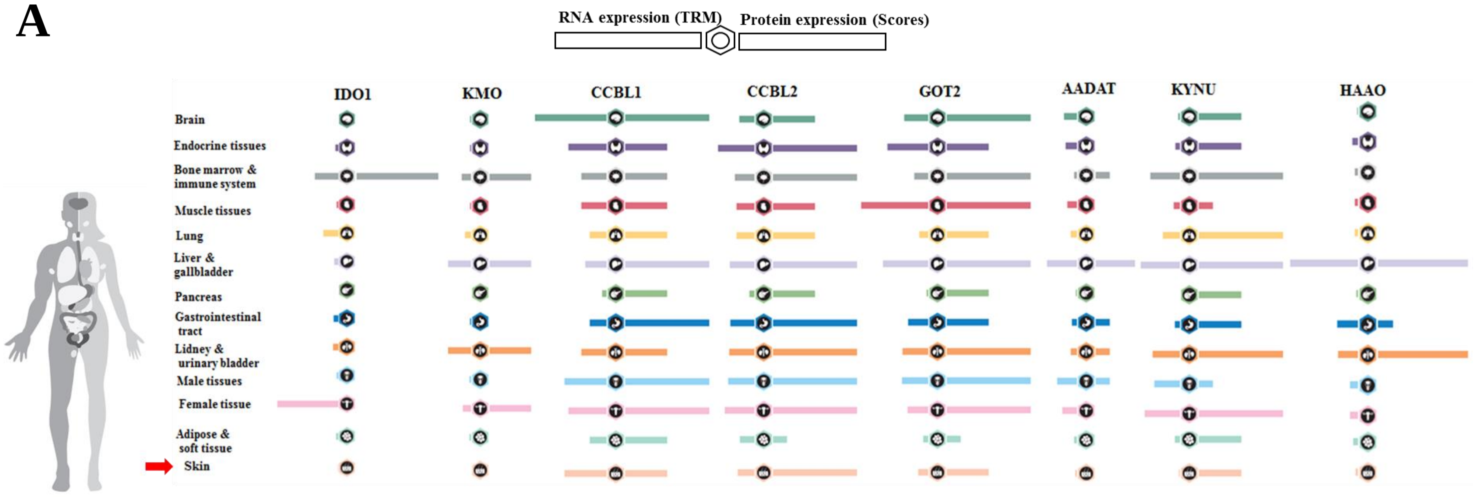

B

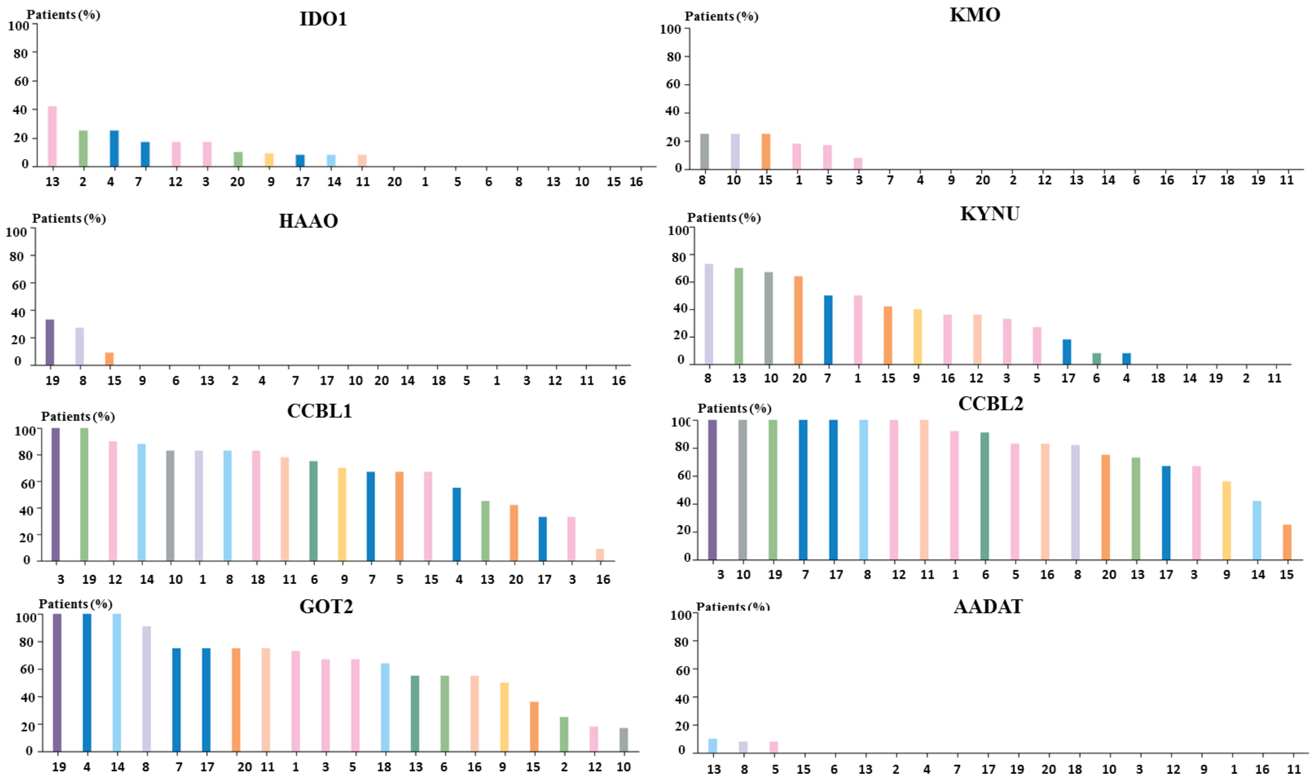

C

Protein expression	Healthy	Melanoma
**CCBL2**	>75	>75
**GOT2**	<25%	75%-25%
**CCBL1**	75%-25%	>75%
**KYNU**	75%-25%	<25%
**AADAT**	N/A	N/A
**HAAO**	N/A	N/A
**IDO1**	N/A	<25%
**IDO2**	N/A	N/A
**KMO**	N/A	N/A
**TDO2**	N/A	<25%

Supplemental Fig. S2. Associations between kynurenine pathway related gene expression and melanoma.(A), KMO, KATs (CCBL1, CCBL2, GOT2 and AADAT) and KYNU related RNA and protein expression in different normal tissues (Fantom5 project) Data from The Human Protein Atlas portal show CCBL1and CCBL2 expression is higher in normal skin tissues compared to KMO, KYNU, GOT2 and AADAT. Image available from proteinatlas.org. (B), KMO, KATs (CCBL1, CCBL2, GOT2 and AADAT) and KYNU related protein expression in different cancers. The cancer types are color-coded according to which type of organ the cancer originates from. For each cancer, plot shows the fraction of patients (maximum 12 patients) with high and medium protein expression level and white bar indicates the low or not identified protein expression results. Data from The Human Protein Atlas portal show CCBL1 (7 of 9 patients show high/medium expression), CCBL2 (12 of 12 patients show high/medium expression) and GOT2 CCBL2 (9 of 12 patients show high/medium expression) expression is more prevalent in melanoma compared to KMO, KYNU, HAAO and AADAT and the differences in the expression frequency are significant. Numbers correspond to a cancer type :1; Breast cancer, 2; Carcinoid,3; Cervical cancer, 4; Colorectal cancer, 5; Endometrial cancer, 6; Glioma, 7; Head and neck cancer, 8; Liver cancer, 9; Lung cancer, 10; Lymphoma, 11; Melanoma, 12; Ovarian cancer, 13; Pancreatic cancer, 14; Prostate cancer, 15; Renal cancer, 16; Skin cancer, 17; Stomach cancer, 18; Testis cancer, 19; Thyroid cancer, 20; Urothelial cancer. (C), KMO, KATs (CCBL1, CCBL2, GOT2 and AADAT) and KYNU related protein expression in melanoma patients vs. healthy controls. All proteins in The Human Protein Atlas labeled with immunofluorescence at the single cell level. The protein expression score is a score assigned manually by annotators (https://www.proteinatlas.org/about/assays+annotation#ihk) and for all issue samples, mRNA sequencing was done on Illumina HiSeq2000 and 2500, additional information is found here: https://www.proteinatlas.org/about/assays+annotation#rna).

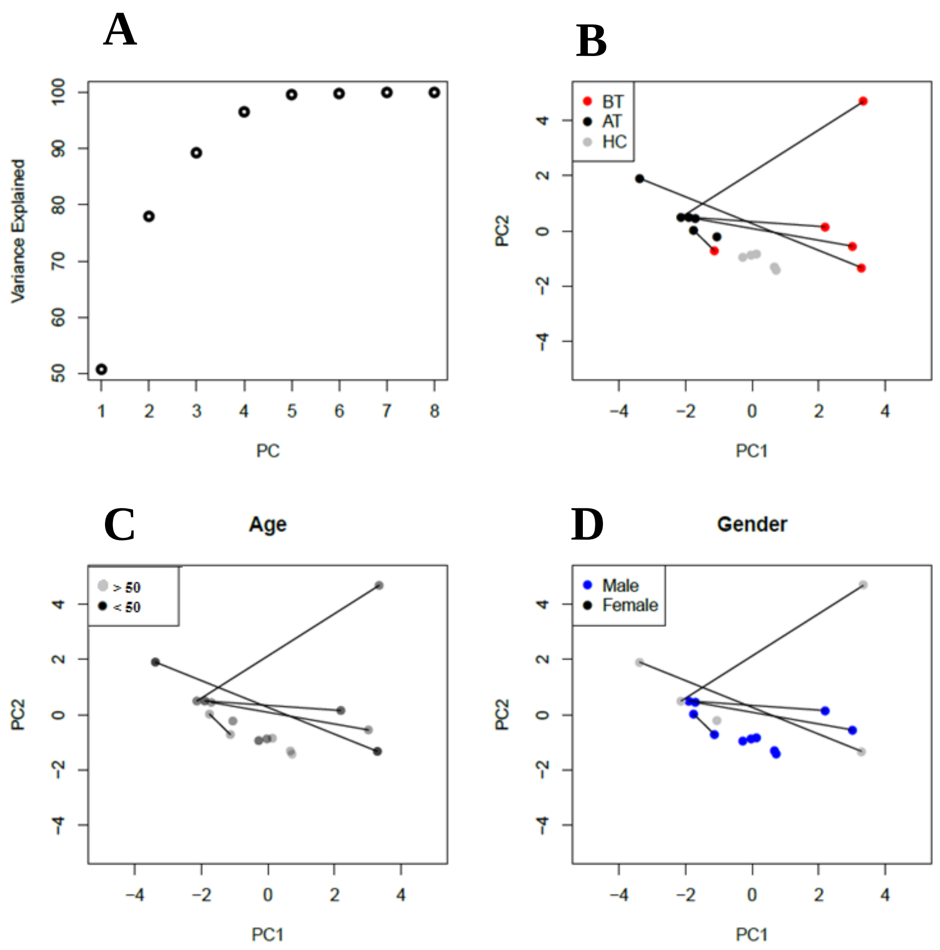

Supplemental Fig. S3. PCA models for unsupervised clustering of healthy control and CMM patient (pre and during treatment)

Principal component analysis of plasma samples from healthy control subjects and CMM patients. (A), this scatter plot shows the first two components explain 80% of the total variance. (B), shows the first (x-axis) versus the second (y-axis) principal component. Samples of healthy individuals are indicated by grey, melanoma samples before treatment by red and CMM patients after treatment by black dots. These three groups can be clearly separated, there were no visible separation of age and gender among samples (C-D).

A

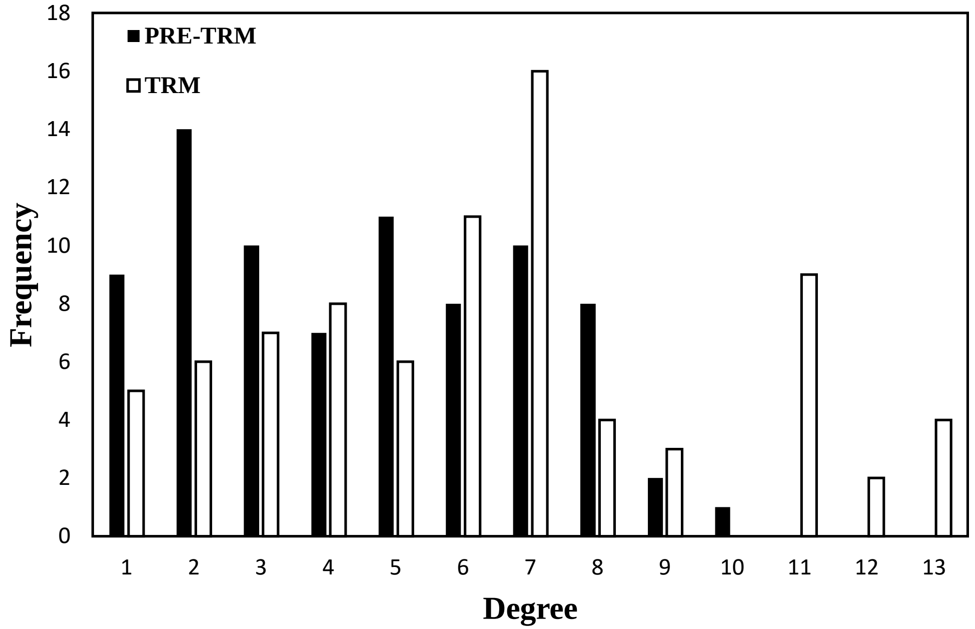

B

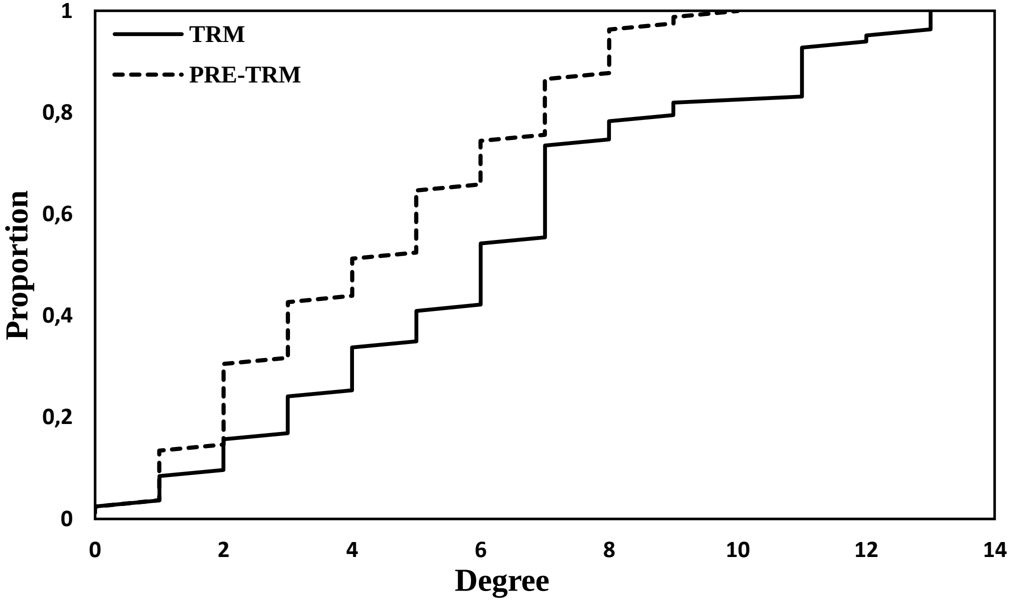

Supplemental Fig. S4. (A), Degree distribution in kynurenine pathway-biomarker networks in PRE and TRM CMM patients. (B), Cumulative degree distribution in biomarker networks PRE and TRM CMM patient. The black (TRM network) and gray lines (PRE-TRM CMM network) indicate the degree values

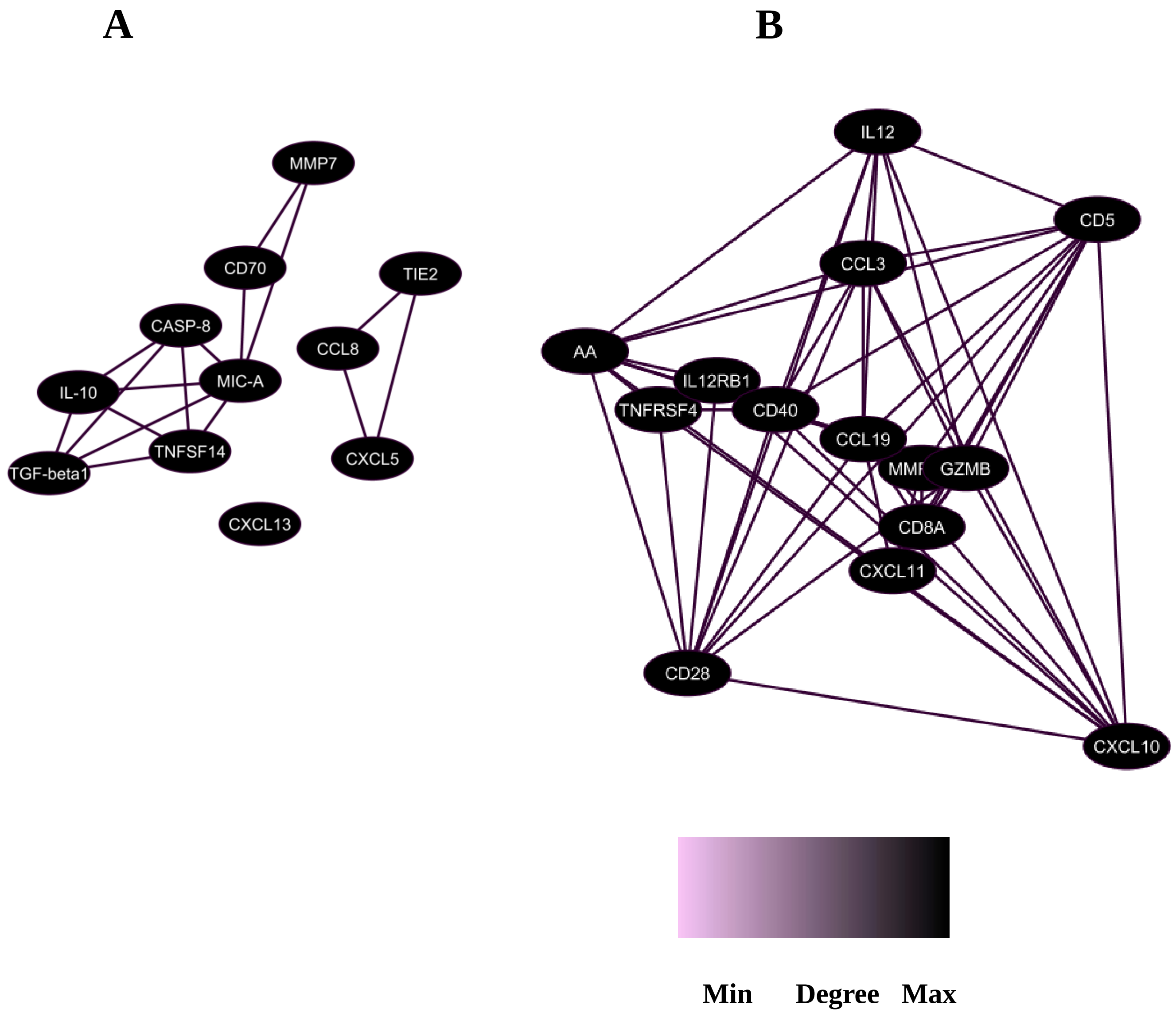

Supplemental Fig. S5. Pattern of CMM plasma protein and anthranilic acid PRE- and TRM groups.

(A), RRE and (B), TRM CMM patients network of highly connected markers[a]. Nodes represents a molecular feature, and an edge specifies a statistically significant Spearman correlation between two markers (nodes).

[a]Highly- connected biomarkers which were at or above the 80th percentile of the degree distribution in the CMM

Supplemental Fig. S6.

Box plots showing the KPMs ratio in healthy individuals compared with CMM patients, (*, p< .05,**, p< .005; Corrected P-Values (Tukey's multiple comparisons test).

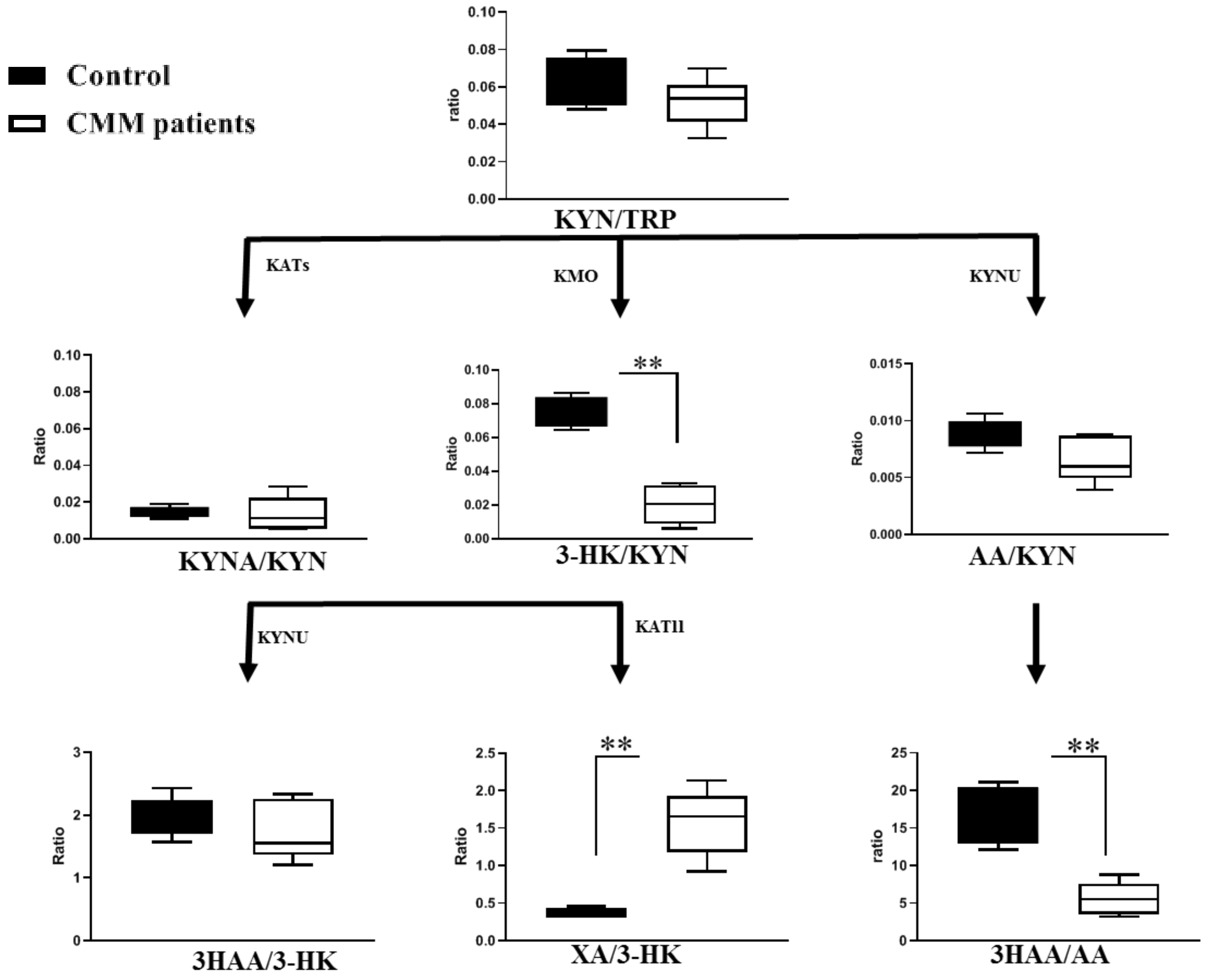

Supplemental Fig. S7. Correlation networks of Immuno-oncology related protein markers and KPMs in CMM plasma samples

(A), Correlation networks between all PRE and (B), TRM CMM patients network on PEA measurement and KPMs, alone. Nodes represents a molecular feature, and an edge specifies a statistically significant Spearman correlation between two markers (nodes).

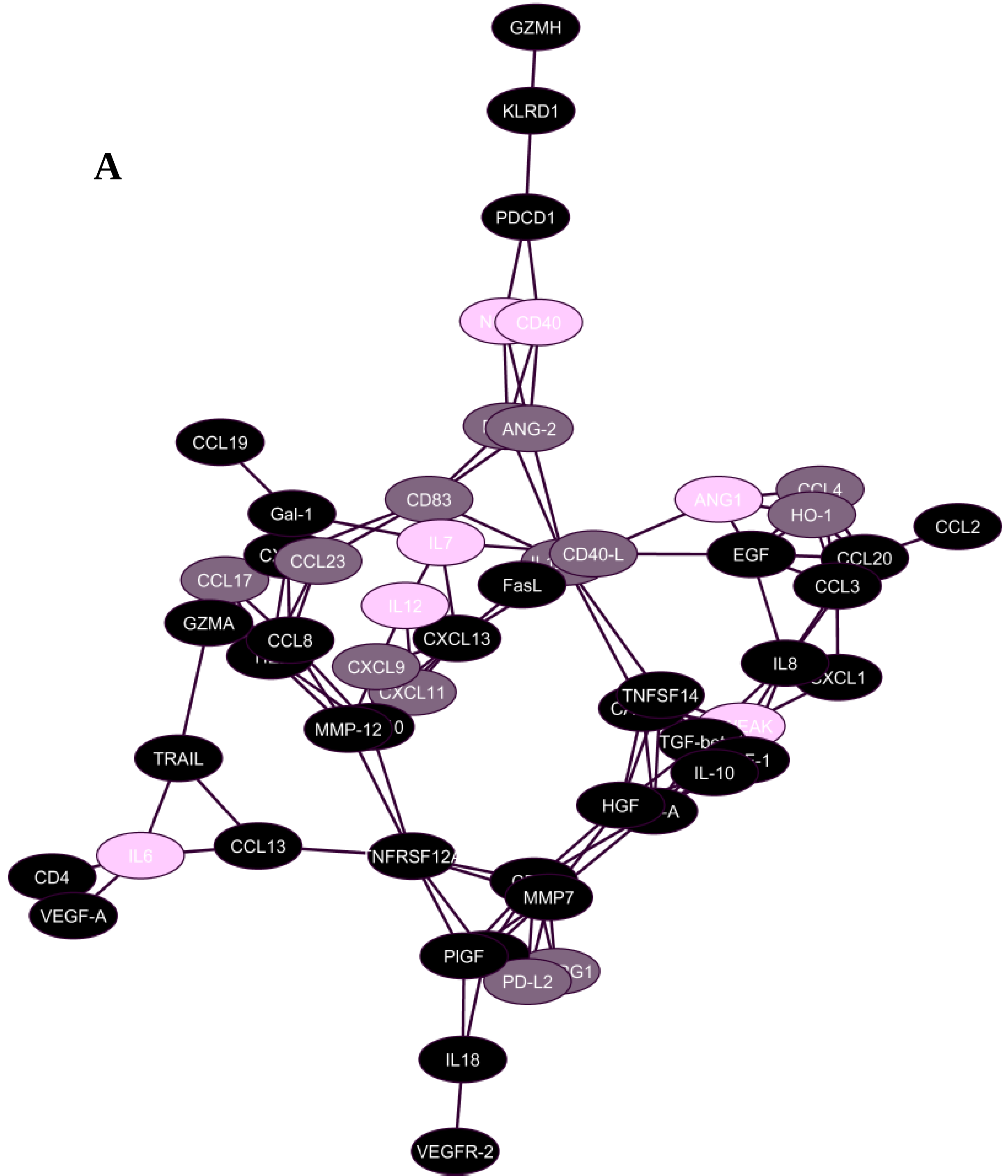

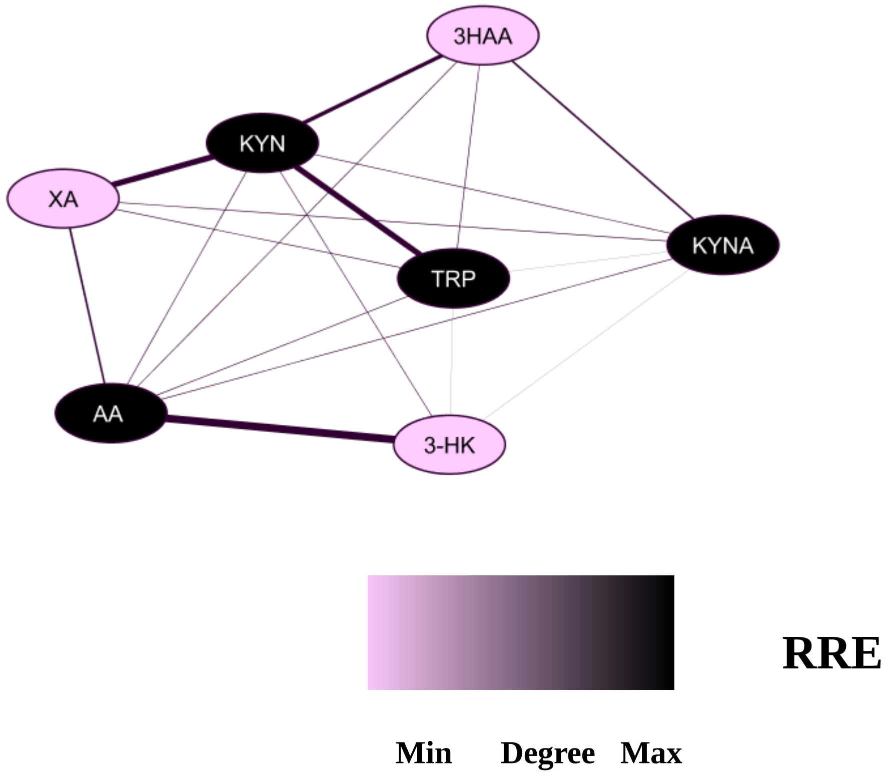

B

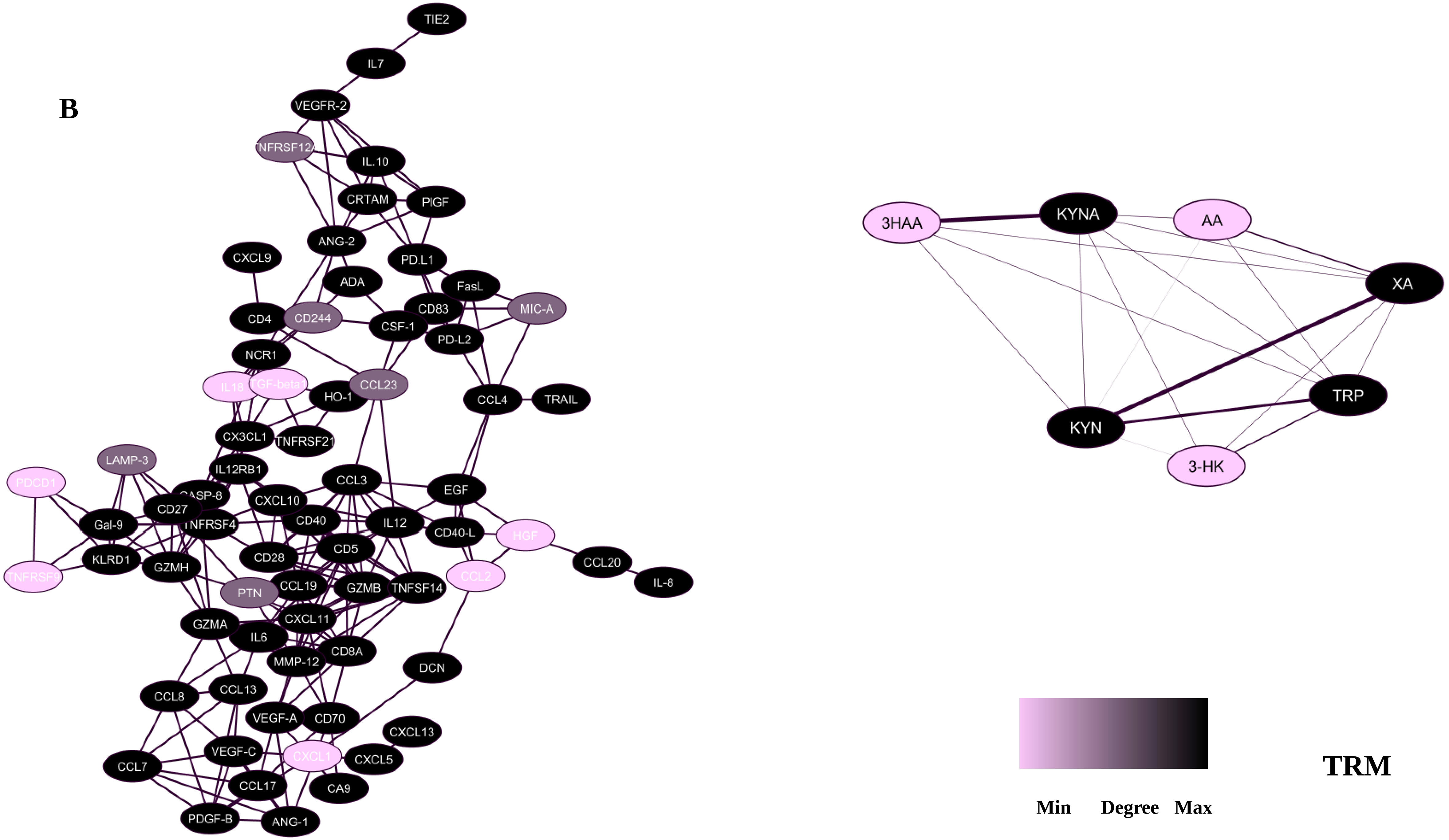
TIE2
IL7
VEGFR-2
TNFRSF12A
IL.10
CRTAM
PlGF
ANG-2
PD.L1
CXCL9
ADA
FasL
CD4
CD244
CD83
MIC-A
CSF-1
PD-L2
NCR1
IL18
TGF-beta1
CCL23
HO-1
CCL4
TRAIL
CX3CL1
TNFRSF21
LAMP-3
IL12RB1
PDCD1
CASP-8
CD27
CXCL10
CCL3
EGF
Gal-9
TNFRSF4
CD40
IL12
CD40-L
HGF
CD28
CD5
CCL20
TNFRSF9
KLRD1
GZMH
CCL2
IL-8
PTN
CCL19
GZMB
TNFSF14
GZMA
CXCL11
IL6
CD8A
MMP-12
DCN
CCL8
CCL13
VEGF-A
CD70
CXCL13
VEGF-C
CXCL1
CXCL5
CCL7
CCL17
CA9
PDGF-B
ANG-1
3HAA
KYNA
AA
XA
TRP
KYN
3-HK
Min
Degree
Max
TRM

A

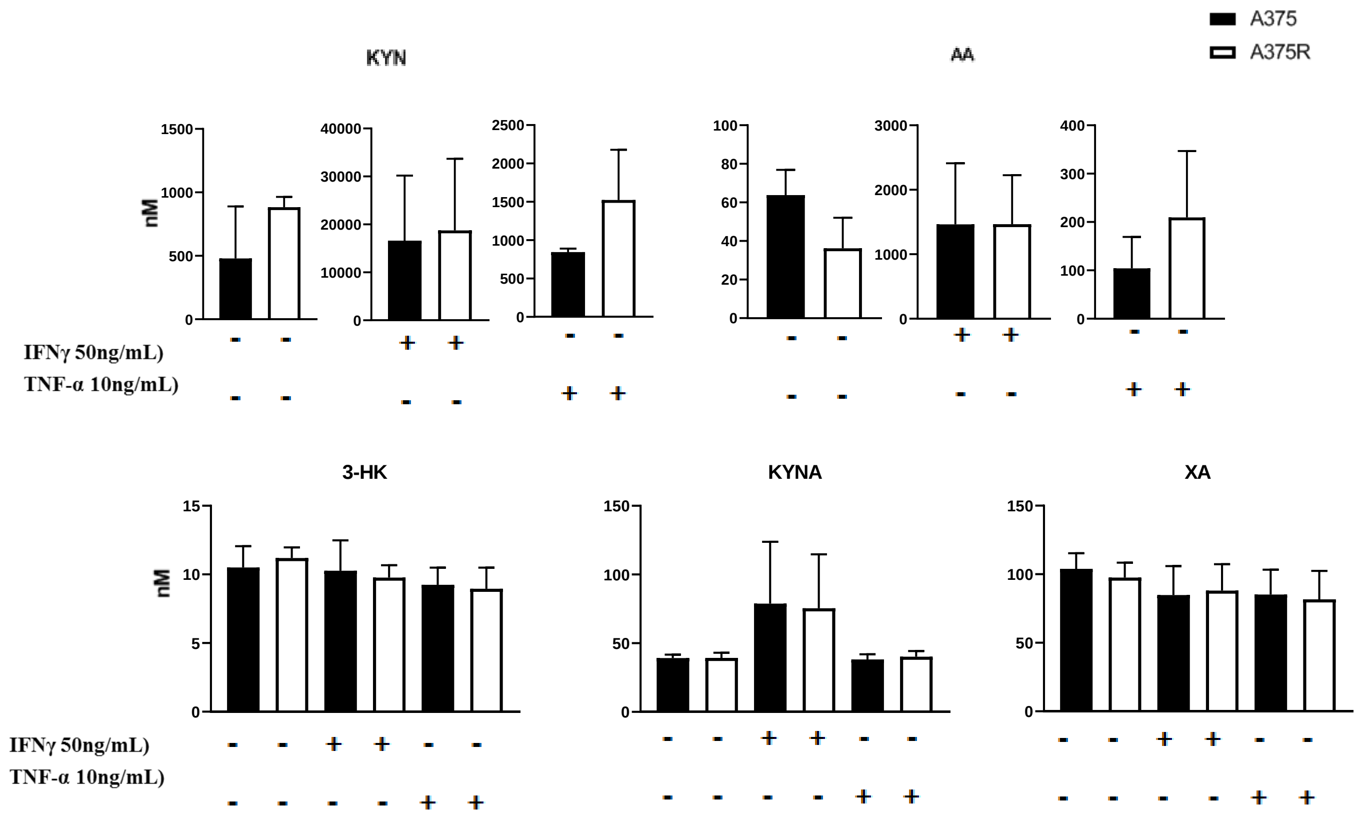

B

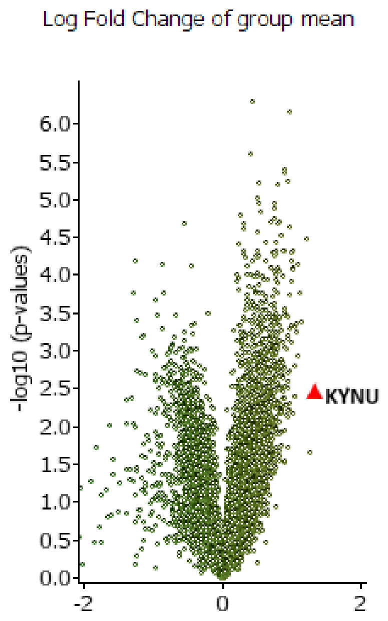

C

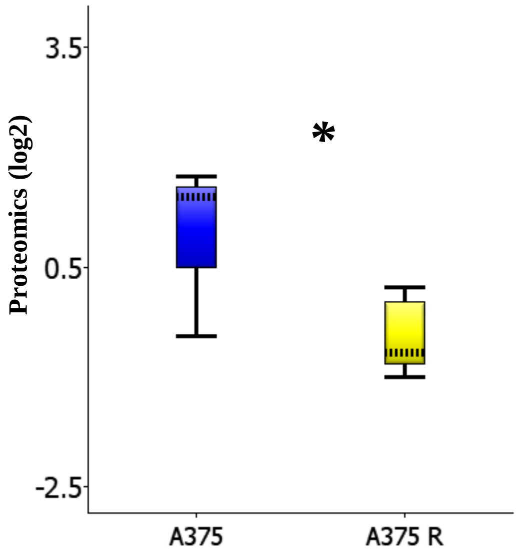

D

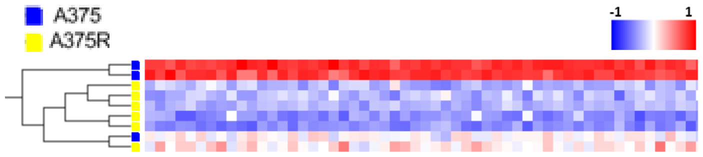

Strongly correlated

SSK1,DIAPH2,HSD14,GSN,PYGL,LDHB,HMGB1,ANXA4,RO60,HSPA8,CAPN 2 ,NFKB1,EEF1B2 ,LAP3,PRDX6,S100A11,TTK MPS1,ATP6V1A ,EPS15,HTT,PITPNB,CCT3 ,STAT5B,RAD23A,PITPNA ,AMPD2,RNASEL,STK4 ,FLII FLIL,BLMH,SMAD2 ,KYNU,NADSYN1,TTC37 ,RAPH1,CCDC91,UBE2Q1,CEP97,UBR1,ANKHD1,TTC39C ,EEF1AKNMT,DYNC2H1,ELMO1,ARHGEF1,GLMN,EXOC4,DCTN5,ASPSCR1,NUCKS1,DOCK5,NIT2,KIF13B,MAN2C1,NDE1,EHD2 , LARS,CHMP2B,SH3BP1,TRAG,LRRFI

**Enriched processes (gene sets)**	**p-value**
Genes encoding cell cycle related targets of E2F transcription factors	2.22E-22
Genes up-regulated through activation of mTORC1 complex	6.4E-7
Genes involved in DNA repair	1.46E-11
Genes important for mitotic spindle assembly	2.28E-9
Genes defining response to androgens	2,00E-07
Genes involved in the G2/M checkpoint, as in progression through the cell division cycle	1.24E-20

Supplemental Fig. S8. KPMs profiling of parental A375 and the MAPKI-resistant sublines, A375R

(A) AA concentration in A375 and A375R, alone and treated with IFNγ (50ng/ml) and TNF-α (10ng/ml) for 48 hours (B), Volcano plot showing differentially regulated proteins in the A375 and A375R cells. A negative log2 value is indicative of downregulation and a positive log2 value shows upregulation of protein expression (C), corresponding box plot showing the KYNU expression in A375 compared with A375R cell lines (D), Heatmap of Spearman correlation coefficient r of highly correlated markers with KYNU targets proteins were listed. (Qlcore Omics Explorer sued to perform these analyses) and, Enriched gene sets from MSigDB for the highly correlated markers with KYNU (>90%) are shown with their indicated p values. (*, p< .05, **, p< .005; Corrected P-Values (Tukey's multiple comparisons test).

A

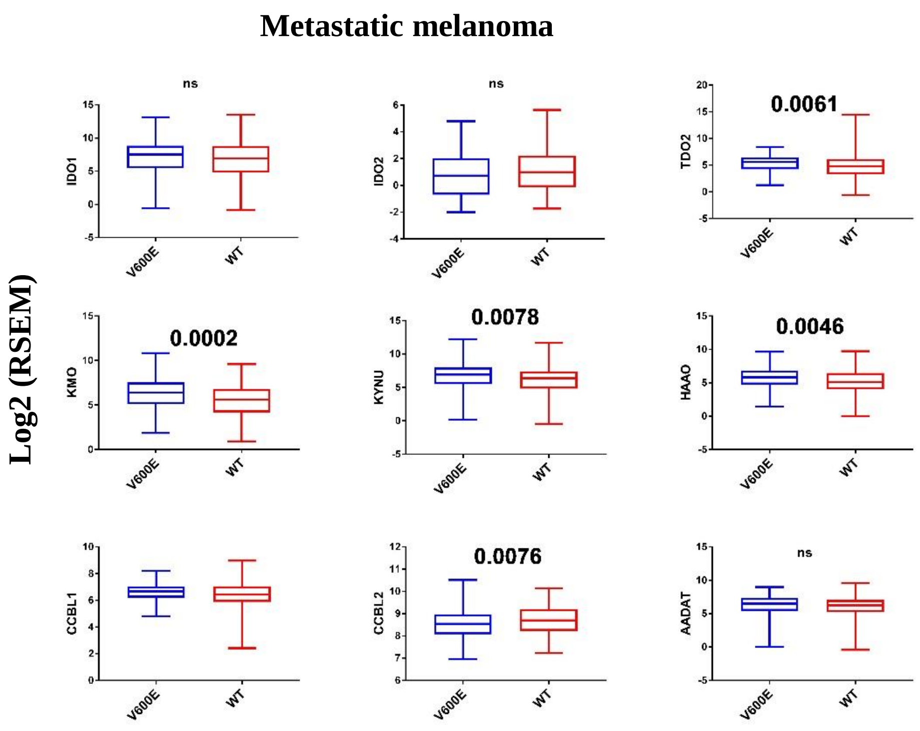

B

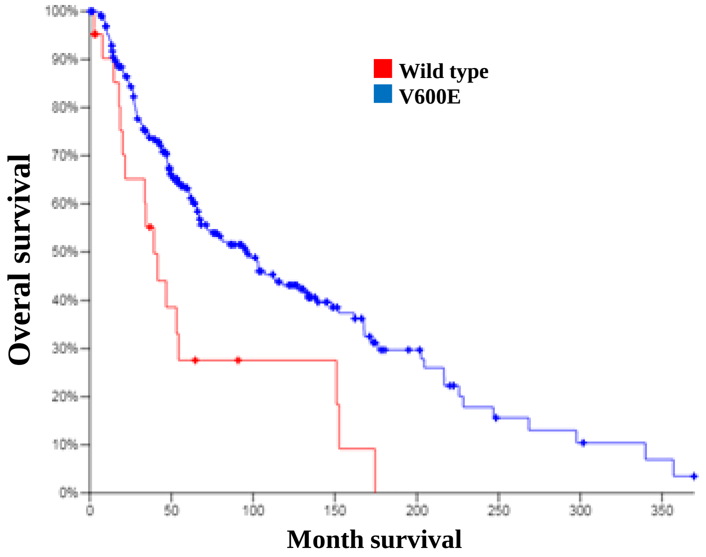

Supplemental Fig. S9. BRAF-mutant CMM shows distinct kynurenine pathway profile

(A), BRAF-mutant CMM retains distinct kynurenine pathway gene expression profile compared to wild type, all samples were assayed by mRNA-seq (39). Gene expression values were represented as RNA-Seq by Expectation Maximization (RSEM), (https://confluence.broadinstitute.org/display/GDAC/Documentation#Documentation-mRNAPipelines). (B), Kaplan–Meier survival plots for BRAF mutation on level 3 Gene expression data of mRNA sequencing on skin cutaneous melanoma (SKCM) were acquired from TCGA that was processed by the Broad Institute’s pipeline (Firehose run “28 January 2016”: doi:10.7908/C11G0KM9) (Supplementary Table S3). Our analysis run was based on the 2016_01_28 data run and included 368 metastatic SKCM samples.

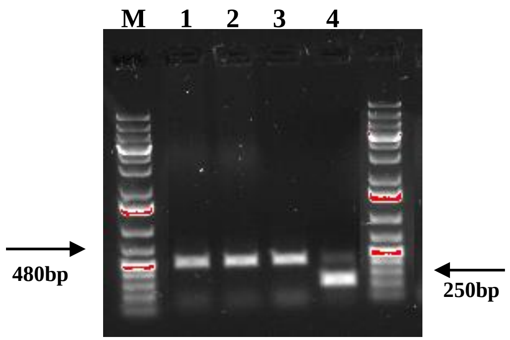

Supplemental Fig. S10 Mycoplasma PCR Detection performed by LookOut Mycoplasma PCR Detection kit, Sigma Aldrich, MP0035. Lane M, amplicon size marker. Bands of 250 bpare characteristic of Mycoplasma, lane 1, negative control; Lanes 2 and 3 are A375 and A375R and cell lines; Lane 6, positive control.
